# Single-Site Experience in the ONSET–OFFSET Study Demonstrates Pharmacodynamic and Pharmacokinetic Advantages of Ticagrelor over Clopidogrel in Patients with Chronic Coronary Syndromes

**DOI:** 10.3390/jcdd13030144

**Published:** 2026-03-20

**Authors:** Heather M. Judge, William A. E. Parker, Robert F. Storey

**Affiliations:** 1 Cardiovascular Research Unit, Division of Clinical Medicine, University of Sheffield, Sheffield S10 2TN, UK; 2 NIHR Sheffield Biomedical Research Centre, Sheffield Teaching Hospitals NHS Foundation Trust, Sheffield S10 2JF, UK

**Keywords:** ticagrelor, platelet aggregation inhibitor, clopidogrel, coronary artery disease

## Abstract

The ONSET–OFFSET study was a multicentre study assessing the pharmacodynamic responses to ticagrelor and clopidogrel in aspirin-treated patients with chronic coronary syndromes. Recent concerns have been raised about the study methodology, including at the single United Kingdom (UK) site in Sheffield. Here, we report data generated at this site along with additional analyses. The UK site recruited 40 out of 123 of the study participants. Platelet P2Y_12_ receptor inhibition was assessed using light transmission aggregometry and whole-blood methodologies. Samples were obtained during the onset and offset dosing periods. Percentage inhibition of platelet aggregation (%IPA) was calculated with (pre-specified) and without (post hoc) truncation of values [0, 100]. Study conduct was monitored by an external contract research organisation. The results from the UK site were concordant with the main study findings. %IPA at 2 h after ticagrelor loading: main study 88%, UK site 91% (truncated), UK site 91% (untruncated). %IPA correlated with other measures of P2Y_12_ inhibition (VerifyNow: *p* < 0.0001; VASP phosphorylation assay: *p* < 0.0001). One patient treated with ticagrelor had >2 h delay in the onset of platelet inhibition associated with co-administration of metformin. The primary endpoint for the offset period was also similar to the main study findings. The UK site data confirm the more rapid onset and offset of inhibitory effects and greater mean levels of platelet inhibition with ticagrelor compared with clopidogrel, regardless of the mode of %IPA data analysis. Study conduct was rigorously monitored in order to demonstrate the integrity and validity of the results. Metformin may delay the onset of action of a ticagrelor loading dose.

## 1. Introduction

The ONSET–OFFSET study was a multicentre, randomised, double-blind study carried out in the USA and UK that studied 123 patients with chronic coronary syndromes [1]. Participants, who were taking aspirin as a single antiplatelet therapy, received either ticagrelor, clopidogrel or placebo for 6 weeks. Platelet function was assessed during the loading and maintenance periods and following the withdrawal of ticagrelor, clopidogrel or placebo to obtain a pharmacodynamic profile of each of the P2Y_12_ inhibitors. The study demonstrated that ticagrelor achieved more rapid inhibition of platelet aggregation (IPA), reaching a near-maximal response by 1 h post-dose (>75% IPA) that was greater than the maximal response achieved with clopidogrel. This greater inhibitory effect of ticagrelor was sustained throughout the maintenance period. Ticagrelor also had a faster offset than clopidogrel with similar IPA at 24 to 48 h following the last maintenance dose. These pharmacodynamic properties of ticagrelor are consistent with its different mechanism of action: ticagrelor is a reversibly binding platelet P2Y_12_ receptor antagonist whose inhibitory effect is dependent on plasma levels of ticagrelor and, to a lesser extent, an active metabolite, both of which have plasma half-lives of approximately 8 to 12 h [2]. The recovery of platelet reactivity following cessation of ticagrelor relies on plasma clearance in ticagrelor and its active metabolite. On the other hand, clopidogrel is a prodrug with wide interindividual variability in the efficiency of conversion to its active metabolite that binds irreversibly to the platelet P2Y_12_ receptor [3]. The recovery of platelet reactivity following cessation of clopidogrel relies on the release of new, uninhibited platelets from the bone marrow, with the life-span of circulating platelets being 7 to 10 days.

Recently, concerns have been raised about the integrity of the ONSET–OFFSET study and questions raised about the methodology, including the conduct of the study at our site [4]. Consequently, we report here more details about the methodology and analyses of the data generated at our site in order to provide more complete details about the conduct of the study and the integrity of the results.

## 2. Methodology

### 2.1. Study Population

The ONSET–OFFSET study was a randomised, double-dummy, parallel-group study in patients with chronic coronary syndromes taking low-dose aspirin 75 to 100 mg once-daily (QD). Further information regarding inclusion and exclusion criteria can be found in the original paper [1]. Participants were randomised to receive a loading dose of ticagrelor 180 mg, clopidogrel 600 mg or placebo followed, respectively, by a maintenance dose of ticagrelor 90 mg twice-daily (BID), clopidogrel 75 mg QD or placebo for 6 weeks. After 6 weeks of maintenance therapy, participants received their final dose of study drug at time 0 of the offset period and were followed for a further 10 days. Of the 123 eligible participants randomised in the global study, 40 were studied at our site between 18 March 2008 and 31 March 2009. The study was conducted in accordance with the Declaration of Helsinki and UK participants provided written informed consent for the study according to a protocol and participant information sheet approved on 4 October 2007 by the UK National Health Service Research Ethics Committee (Fife & Forth Valley Research Ethics Committee, reference number 07/S0501/68).

### 2.2. Blood Sampling

Blood samples were obtained for platelet function testing prior to dosing with study medication and from 0.5 to 24 h after the first loading dose (onset phase) then, after 6 weeks of maintenance therapy, again immediately prior to the last dose of study medication at the start of the offset period and from 2 to 240 h after the last dose.

### 2.3. Platelet Function Assessments

Platelet function was assessed using light transmission aggregometry (LTA) with the agonists adenosine diphosphate (ADP) 20 and 5 μmol/L, collagen 2 μg/mL and arachidonic acid 2 mmol/L, VerifyNow P2Y_12_ assay (Accriva Diagnostics, Inc., San Diego, CA, USA), and vasodilator-stimulated phosphoprotein (VASP) phosphorylation assay (Biocytex VASP kit) (BioCytex, Inc., Marseille, France).

### 2.4. Statistical Analysis

The main study primary endpoint for the onset phase was %IPA determined from final aggregation response to ADP 20 μmol/L at 2 h after loading dose of study medication and the same primary endpoint is used in analyses of the UK site data. The main study primary endpoint for the offset phase was the slope of IPA between 4 and 72 h post-dose and, again, this endpoint is used in this UK site data analysis. Results obtained for light transmission aggregometry are presented as %IPA where PAb and PAt are the responses at baseline and post-dosing, respectively:

$$IPA\left(\%\right)=\frac{PAb-PAt}{PAb}\times \;100$$

The protocol pre-specified that the estimate of IPA would be restricted to a closed limit [0, 100] and that any data falling outside this range would be truncated to the appropriate limit, i.e., 0% for negative values and 100% for values greater than 100%. In view of the suggestion that truncating the data may influence the outcomes of the study [4], here we present both the truncated %IPA (%IPA truncated), as previously published in the main study, and new analysis of the %IPA without any truncation (%IPA untruncated).

High platelet reactivity (HPR) was assessed using previously established thresholds to determine the numbers (percentages) of participants with LTA maximum aggregation response > 59%, VerifyNow PRU > 208 or VASP PRI > 50% [3,5,6].

Correlation between the different platelet function assay results was determined using simple linear regression.

Statistics for the current analyses were performed using GraphPad PRISM software version 10.0.

### 2.5. Study Monitoring, Training and Consumable Supplies

The study was monitored by ICON Clinical Research PLC (Reading, UK). All staff were experienced on all techniques used in the study and underwent additional study-specific training prior to commencing study-related activities.

Laboratory reagents were supplied by ICON PLC. Concerns were raised about VerifyNow P2Y_12_ test results being obtained prior to evidence of study-specific instruments and test cartridges arriving at our site [4]. At this time, the laboratory was engaged in the PLATO platelet function substudy [7] and the RESPOND study [8] and had both VerifyNow instruments and VerifyNow P2Y_12_ tests in stock. Email confirmation of stock levels with the monitor on 13 May 2008 indicated that there were 33 boxes of P2Y_12_ cartridges, each containing 25 cartridges.

## 3. Results

### 3.1. Patient Population and Monitoring

**Figure 1:**
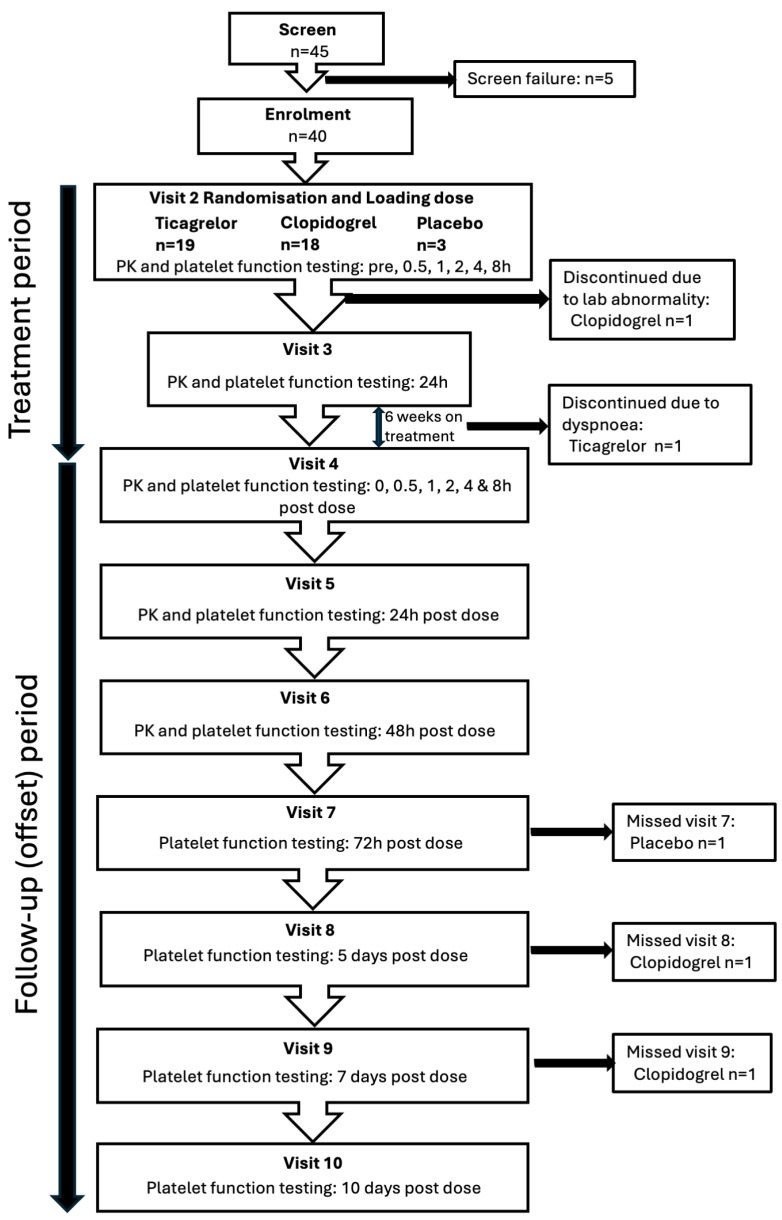
Participant flow and visits.

Forty participants were randomised in the ONSET–OFFSET study at our site: 19 participants received ticagrelor, 18 clopidogrel and 3 placebo (Figure 1). The monitoring contract research organisation performed 55 site visits between 27 November 2007 and 10 March 2009 to oversee the conduct of the study and confirm integrity of the data.

### 3.2. Primary Outcome for Onset Phase

**Figure 2:**
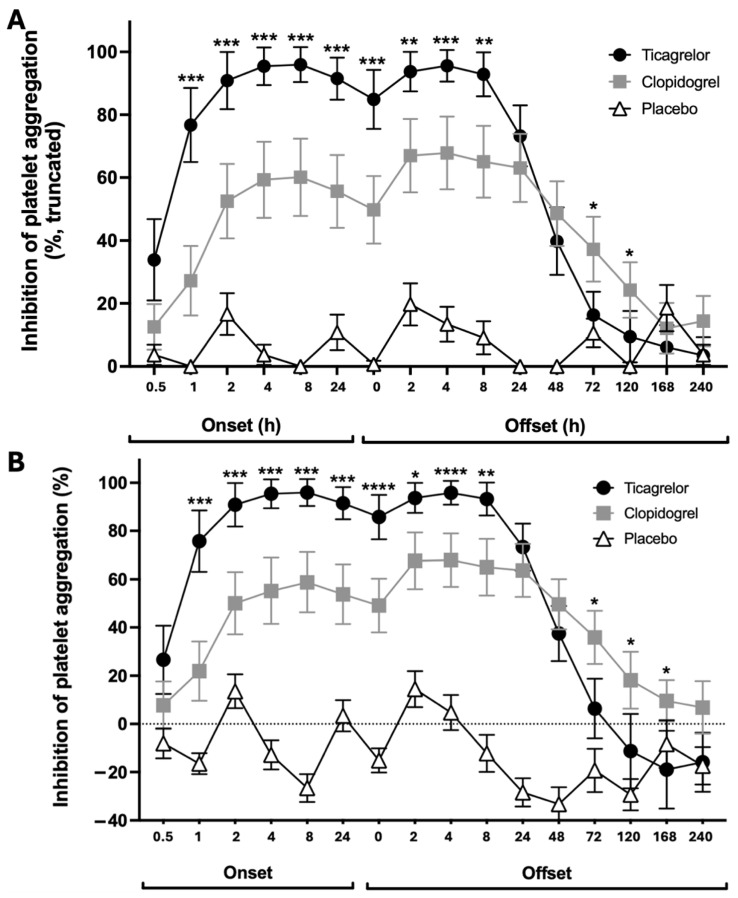
Inhibition of platelet aggregation assessed by light transmittance aggregometry. Inhibitory effects of ticagrelor, clopidogrel and placebo on the final platelet aggregation response to adenosine diphosphate 20 μmol/L, showing percentage inhibition of platelet aggregation (**A**) with truncation of values at 0 and 100%, and (**B**) without truncation of values. Data are mean, error bars indicate SEM. * *p* < 0.05; ** *p* < 0.01; *** *p* < 0.001; **** *p* < 0.0001.

UK site data with pre-specified truncation of values (Figure 2A) as well as without truncation (Figure 2B) showed a more rapid onset of platelet inhibition and greater peak %IPA with ticagrelor compared with clopidogrel. Our site data were broadly comparable to the overall study results (Table 1). %IPA was higher at 1 h in the participants taking ticagrelor (*p* = 0.0001) and at all time points during the onset period (*p* < 0.05), regardless of whether or not there was truncation of the values (Figure 2).

Five out of 19 participants randomised to receive ticagrelor and two out of 18 participants randomised to receive clopidogrel had <50% maximal and final aggregation responses to ADP 20 μmol/L at baseline, prior to study drug administration, leading to some negative untruncated %IPA values during the onset period. This suggests some platelet desensitisation due to blood sampling and/or centrifugation, which may have led to underestimation of the rate of the onset of action of the drugs.

### 3.3. Primary Outcome for Offset Phase

At the end of the maintenance period, IPA with ticagrelor was significantly higher than with clopidogrel (Figure 1) (%IPA: *p* < 0.0001; %IPA truncated: *p* = 0.0002). Truncated and untruncated UK %IPA data during the offset period showed a similar offset of ticagrelor and clopidogrel as demonstrated previously in the main study (Table 2). IPA did not differ significantly between the groups at 24 and 48 h after the last dose. The ticagrelor group had significantly lower %IPA (truncated or untruncated) than the clopidogrel group at 72 and 120 h after the last dose (Figure 2).

### 3.4. VerifyNow P2Y_12_ and VASP Phosphorylation Assays

**Figure 3:**
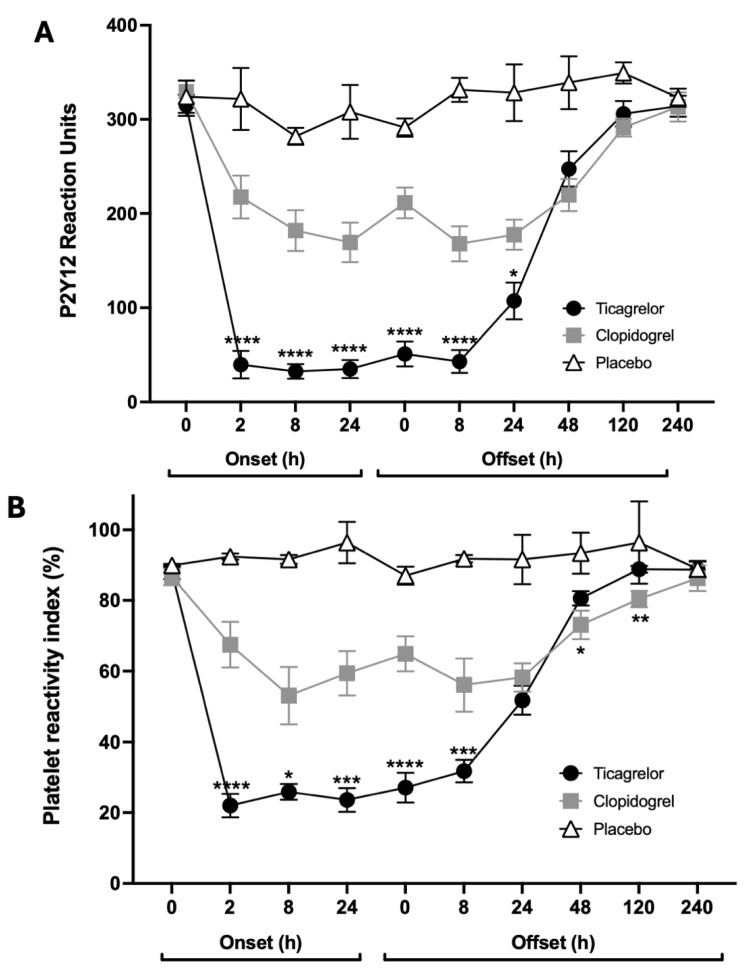
Platelet reactivity assessed by VerifyNow P2Y12 and VASP phosphorylation assays. Onset, maintenance and offset of inhibitory effects of ticagrelor, clopidogrel and placebo on platelet reactivity determined as (**A**) P2Y_12_ reaction units (PRU) and (**B**) percentage platelet reactivity index (PRI). Data are mean platelet reaction units, error bars indicate SEM. * *p* < 0.05; ** *p* < 0.01; *** *p* < 0.001; **** *p* < 0.0001.

VerifyNow PRU values demonstrated consistent findings with the LTA data, showing a more rapid onset of action, lower platelet reactivity after loading and during maintenance therapy, and a more rapid offset of effect with ticagrelor compared with clopidogrel (Figure 3A). The VASP PRI data were concordant with these findings (Figure 3B).

### 3.5. High Platelet Reactivity (HPR) Analyses

**Figure 4:**
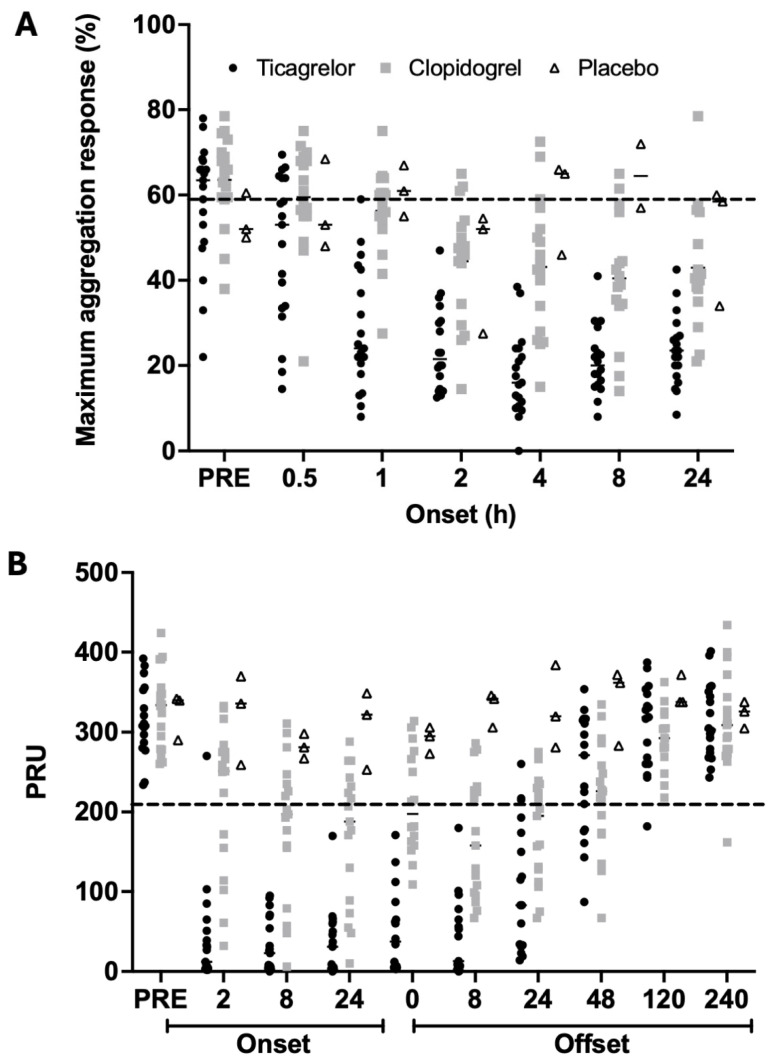

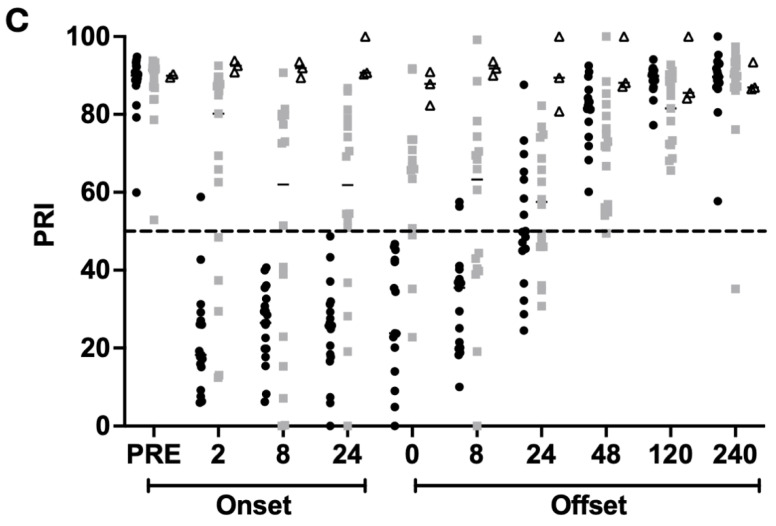
Individual participant results demonstrating proportions with high platelet reactivity (HPR). Data show individual participant results and median (short horizontal lines) according to randomised treatment group for (**A**) maximum percentage platelet aggregation response using light transmittance aggregometry and HPR threshold of >59% (dashed line), (**B**) VerifyNow P2Y_12_ assay showing P2Y_12_ reaction units (PRU) and HPR threshold of >208 PRU (dashed line), and (**C**) VASP phosphorylation assay showing platelet reactivity index (PRI) truncated to [0, 100] and HPR threshold of >50% (dashed line).

A substantial proportion of participants in all the cohorts had maximum LTA response to ADP 20 μmol/L less than 59% at baseline, suggesting LTA is less suited to HPR analyses than the VerifyNow and VASP whole-blood assays, which showed HPR in all participants prior to the loading dose of study medication (Figure 4).

**Figure 5:**
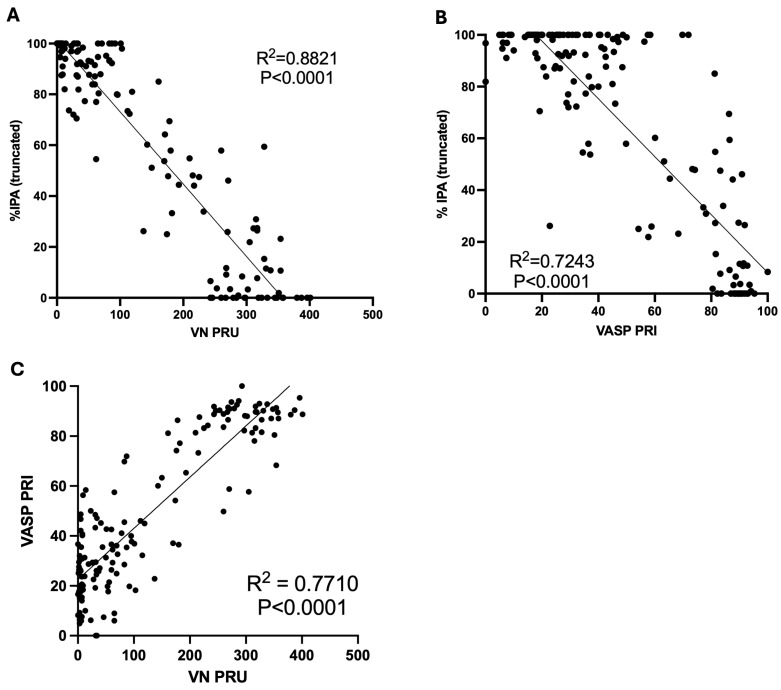
Correlations between different platelet function measurement values. Correlation between (**A**) percentage inhibition of platelet aggregation (%IPA), using light transmittance aggregometry with truncation of values at 0 and 100%, and VerifyNow P2Y_12_ reaction units (VN PRU); (**B**) percentage inhibition of platelet aggregation (%IPA), using light transmittance aggregometry with truncation of values at 0 and 100%, and vasodilator-associated phosphoprotein platelet reactivity index (VASP PRI); and (**C**) VN PRU and VASP PRI. R^2^ and *p* values were determined using simple linear regression (GraphPad Prism v10).

There was 5% HPR (PRU > 208) at 2 h after the ticagrelor loading dose compared to 65% in the clopidogrel group (Table 3; Figure 4B). Similarly, the VASP PRI data showed 6% HPR after ticagrelor at 2 h compared to 72% after clopidogrel (Figure 4C). There were moderate-to-good correlations between PRU values, PRI values and %IPA values determined by LTA (Figure 5).

The single patient with HPR at 2 h after ticagrelor, determined by both VerifyNow and VASP assays, subsequently achieved a PRU of 54 and a PRI of 18% at 8 h post-dose and was not receiving any concomitant opiate medication but had diabetes mellitus treated with metformin; the suppression of platelet reactivity occurred by 4 h post-dose according to the LTA findings: final LTA response to ADP 20 μmol/L was 42% at 2 h and 5% at 4 h post-dose.

## 4. Discussion

The UK site data for the ONSET–OFFSET study are concordant with the overall study results, demonstrating a more rapid onset of action, consistently greater mean %IPA and a more rapid offset of effect for ticagrelor compared with clopidogrel. Truncating the %IPA data did not influence the primary study findings since a similar level of inhibition was observed when the %IPA data were calculated as either truncated, as per the original study publication, or untruncated.

We can confirm that the UK analyses were conducted by experienced and trained staff and the site and study outputs were rigorously monitored by an experienced contract research organisation. It is well recognised that the LTA method is limited by artefacts that may arise as a result of the centrifugation process to produce platelet-rich plasma and the impact of hydration status and dietary intake, particularly of fatty foods that cause lipaemia and affect the optical density of plasma [9]. Whilst the protocol stipulated dietary restriction prior to blood sampling for LTA, there was still evidence of platelet desensitisation in the baseline platelet-rich plasma samples of some participants, reflected by maximum aggregation values of less than 50%. This reflects a limitation of using IPA derived from LTA in clinical studies of antiplatelet drugs and explains the move to whole-blood methods that has occurred over the last 17 years since the ONSET–OFFSET study was conducted. Indeed, the results of the whole-blood assays (VerifyNow P2Y_12_ assay and VASP PRI) in our study provide reassurance about the conclusions based on the LTA results, reinforcing the findings of ticagrelor’s pharmacodynamic advantages over clopidogrel. Since these whole-blood assays do not involve centrifugation of blood prior to analysis, they avoid the potential for platelet desensitisation seen with the LTA methodology. Furthermore, they are both designed to optimise the detection of platelet P2Y_12_ inhibition and demonstrate the profound and consistent reduction in P2Y_12_-mediated platelet reactivity seen with ticagrelor compared to the moderate and variable reduction seen with clopidogrel.

The results of the ONSET–OFFSET study are supported by many other studies that have assessed the pharmacological properties of ticagrelor and/or clopidogrel that demonstrate more consistent antiplatelet effects of ticagrelor and a lower incidence of high platelet reactivity [6,10,11,12]. The pharmacological advantages of ticagrelor over clopidogrel explain the greater clinical efficacy of ticagrelor seen in the pivotal PLATO study [7,13,14,15,16,17], as well as the more frequent incidence of spontaneous bleeding events. The more rapid offset of effect of ticagrelor explains why its greater level of platelet inhibition during maintenance therapy did not lead to more perioperative bleeding in PLATO [7,16,17].

As a novel finding, we identified one patient treated with ticagrelor who had a slower onset of action of the loading dose, taking up to 4 h for full platelet P2Y_12_ inhibition to occur. This patient was treated with metformin, which has recently been associated with delayed gastric emptying [18,19], thus indicating a potential adverse impact of diabetes medications on the speed of the onset of oral platelet P2Y_12_ inhibitors in patients presenting with acute myocardial infarction.

## 5. Conclusions

The UK site data for the ONSET–OFFSET study demonstrate the rigorous conduct of the study and support the findings of the main study that ticagrelor has a more rapid onset of action, more consistent inhibitory effect during maintenance therapy, and a more rapid offset of action compared with clopidogrel. These conclusions are not affected by truncation of the %IPA results to compensate for the effects of blood centrifugation and are supported by whole-blood assays that are optimised for sensitivity to platelet P2Y_12_ receptor inhibition. Further research is warranted to assess any negative impact of metformin on the speed of the onset of action of oral platelet P2Y_12_ inhibitors.

## Figures and Tables

**Table 1:** Percentage inhibition of the final platelet aggregation response to ADP 20 μmol/L at 2 h post-loading dose in the overall study and at the UK site.

% Inhibition of Final Aggregation (20 μmol/L) 2 h	Ticagrelor		Clopidogrel		*p*
Mean ± SD	%IPA	*n*	%IPA	*n*	
Overall study—truncated	88 ±15	57	38 ± 33	54	<0.0001
UK site—truncated	91 ± 18	19	53 ± 34	18	0.0003
UK site—untruncated	91 ± 18	19	50 ± 39	18	0.0003

The UK data are presented as either truncated [0, 100] or untruncated data. Data are mean ± SD. Significance was assessed using Mann–Whitney U test.

**Table 2:** Slope for the offset period for the main study and the truncated and untruncated UK data.

Final Extent 20 μmol/L ADP		Slope [4–72 h]	Y Intercept
Main study	Ticagrelor	−1.04	94.00
UK (IPA truncated)	Ticagrelor	−1.21	101.1
UK (IPA untruncated)	Ticagrelor	−2.89	90.98
Main study	Clopidogrel	−0.48	71.84
UK (IPA truncated)	Clopidogrel	−0.45	70.40
UK (IPA untruncated)	Clopidogrel	−0.46	70.88

UK results were calculated using linear regression between 4 and 72 h post-dose.

**Table 3:** Percentage of participants with high platelet reactivity assessed using the VerifyNow P2Y_12_ and VASP phosphorylation assays.

Visit (h)		VerifyNow PRU > 208		VASP-P Assay PRI > 50%	
		Ticagrelor	Clopidogrel	Ticagrelor	Clopidogrel
ONSET	0	100% (17/17)	100% (18/18)	95% (18/18)	100% (18/18)
	2	5% (1/19)	65% (11/17)	6% (1/17)	72% (13/18)
	8	0% (0/19)	47% (8/17)	0% (0/19)	59% (10/17)
	24	0% (0/19)	29% (5/17)	0% (0/18)	76% (13/17)
OFFSET	0	0% (0/16)	44% (7/16)	0% (0/15)	80% (12/15)
	8	0% (0/17)	41% (7/17)	12% (2/17)	59% (10/17)
	24	18% (3/17)	41% (7/17)	47% (8/17)	59% (10/17)
	48	71% (12/17)	65% (11/17)	100% (17/17)	94% (16/17)
	120	94% (16/17)	100% (16/16)	100% (16/16)	100% (16/16)
	240	100% (18/18)	100% (18/18)	100% (17/17)	94% (15/16)

Data show % high platelet reactivity and the proportion of the total number of participants per visit. PRI: platelet reactivity index; PRU: P2Y_12_ reaction units; VASP-P: vasodilator-stimulated phosphoprotein phosphorylation.

## Data Availability

The original contributions presented in this study are included in the article. Further inquiries can be directed to the corresponding author.
